# Development of a 3D Anthropomorphic Phantom Generator for Microwave Imaging Applications of the Head and Neck Region

**DOI:** 10.3390/s20072029

**Published:** 2020-04-04

**Authors:** Ana Catarina Pelicano, Raquel C. Conceição

**Affiliations:** 1 Faculdade de Ciências da Universidade de Lisboa, Campo Grande, 1749-016 Lisbon, Portugal; acpelicano@fc.ul.pt; 2 Instituto de Biofísica e Engenharia Biomédica, Faculdade de Ciências da Universidade de Lisboa, Campo Grande, 1749-016 Lisbon, Portugal

**Keywords:** anthropomorphic phantoms, head and neck modelling, radar-based microwave imaging, cervical lymph nodes

## Abstract

The development of 3D anthropomorphic head and neck phantoms is of crucial and timely importance to explore novel imaging techniques, such as radar-based MicroWave Imaging (MWI), which have the potential to accurately diagnose Cervical Lymph Nodes (CLNs) in a neoadjuvant and non-invasive manner. We are motivated by a significant diagnostic blind-spot regarding mass screening of LNs in the case of head and neck cancer. The timely detection and selective removal of metastatic CLNs will prevent tumor cells from entering the lymphatic and blood systems and metastasizing to other body regions. The present paper describes the developed phantom generator which allows the anthropomorphic modelling of the main biological tissues of the cervical region, including CLNs, as well as their dielectric properties, for a frequency range from 1 to 10 GHz, based on Magnetic Resonance images. The resulting phantoms of varying complexity are well-suited to contribute to all stages of the development of a radar-based MWI device capable of detecting CLNs. Simpler models are essential since complexity could hinder the initial development stages of MWI devices. Besides, the diversity of anthropomorphic phantoms resulting from the developed phantom generator can be explored in other scientific contexts and may be useful to other medical imaging modalities.

## 1. Introduction

Head and neck cancer is a generic term referring to a large group of diseases characterized by the abnormal growth of epithelial cells in the oral and nasal cavities, paranasal sinuses, salivary glands, pharynx and larynx, and also their ability to invade nearby tissues through lymphatic and blood vessels [1]. In 2018, this cancer reported 887,659 worldwide new cases, with over 51% death incidence [2]. The assessment of the Lymph Nodes (LNs) within the head and neck region—also referred to as cervical lymph nodes—is critical for cancer staging and consequently, for therapy decisions. The state-of-the-art Tumor–Nodes–Metastasis (TNM) staging system used to clinically diagnose head and neck cancers often involves the excision and histopathology analysis of the Cervical LNs (CLNs) closest to the primary site of the tumor [3]—the sentinel nodes—which are the ones with highest probability of being targeted by cancer cells. Depending on the type of head and neck tumor, different CLNs from levels I-VI may be removed [4]. The excision of the LNs for cancer staging commonly results in the removal of healthy LNs, which has a negative impact on patients’ health and quality of life, and it is costly to healthcare systems due to the patients’ need for continuous monitoring and follow-up treatments [5,6]. With the removal of too many CLNs, patients’ physical recovery often becomes a slower process and the associated morbidity risk increases, namely their speech and swallowing may be hampered, and the physical appearance is oftentimes deformed. In addition, the removal of healthy LNs increases the risk of infection and lymphedema [7]. Other imaging modalities have been reported to attempt CLNs screening, such as ultrasound, Computed Tomography (CT), Positron Emission Tomography (PET) and Magnetic Resonance Imaging (MRI), but none have provided a definitive satisfactory neoadjuvant diagnosis to assess whether the cancer has spread to neighboring LNs [8].

As a result of the lack of alternatives to accurately assess CLNs, radar-based MWI presents itself as a promising screening modality. This technique uses microwave frequency signals and the dielectric contrast between different tissues for detecting shallow body structures or regions of interest, which ultimately can help in the detection of the CLNs [9]. In addition to being a comfortable and non-invasive imaging modality, it is also portable, low-cost, user-independent, and uses low power. 

For the development process of MWI systems, numerical and physical phantoms capable of mimicking the electromagnetic behavior of the body head and neck regions, at microwave frequencies, are needed. In the context of the head region, Scapaticci et al. [10] developed a planar numerical model of the human head which consisted of five layers, with different dielectric properties and lengths, concerning skin tissue, fat tissue, cortex bone, CerebroSpinal Fluid (CSF) and brain tissue. A single-pole Cole–Cole model was used to evaluate the behavior of the dielectric properties of biological tissues over a frequency range from 0.1 to 10 GHz. More recently, Fhager et al. [11], have added more realism to head phantoms in order to create a more realistic simulation environment. They based their research on an existing anatomical tissue model of a healthy subject from BrainWeb [12]. This model includes the main tissues of the head such as CSF, blood, muscle, grey and white matter, fat and skull. They used the Debye model to describe the dispersive behavior of the different tissues’ dielectric properties in their Finite-Difference Time-Domain (FDTD) model.

Regarding physical head phantoms, Bjelogrlic et al. [13] contributed to the state-of-the-art by designing a spherically stratified model in which every concentric layer between the brain and the background medium represented a particular human head tissue. They printed a multilayered spherical structure using fused deposition modeling technology with white Acrylonitrile Butadiene Styrene (ABS) plastic. Each shell with a 2 mm thickness was filled with liquids mimicking the main human head tissues, such as brain tissue, CSF, cortical bone, fat and skin. Both numerical and physical phantoms allowed representations of the head region with more or less detail.

Most reported head and neck physical phantoms developed to date were used in the context of radiation dosimetry, in which they must be able to represent the biological tissues regarding their x-ray absorption and scattering properties, and their dimensions, thicknesses and depths. Brand et al.’s [14] phantom considered only the bone tissue of the human skull and cervical vertebrae, soft tissues for the interior of the phantom and facial contours and also, air passages in the oral and nasal cavities, paranasal sinuses, pharynx, and larynx. Molineu et al. [15] designed a head and neck phantom for intensity-modulated radiation therapy which consists of an anatomically realistic outer plastic shell for the contour of the head and neck, imageable targets with radiation dosimeters, and a remaining volume of water.

There are fewer head and neck models reported in literature, mostly developed in the context of hyperthermia. Paulides et al. [16] developed an elliptical shape neck with a homogenous muscle and some major structures such as trachea and spine. These structures were modeled based on semi-3D approximations from CT scans. Merunka et al. [17] adapted and simplified a neck phantom from the whole human body phantom of [18]. The simplified phantom consisted of a cylindrical shape with a diameter of 55 mm and 100 mm of height, with four structures: skin, muscle, cervical vertebrae, and larynx with tumor tissues.

In the context of radar-based MWI, we developed a phantom generator of 3D, anatomically realistic electromagnetic head and neck models derived from MRI exams, capable of describing the realistic dielectric properties of biological tissues for microwave frequencies from 1 to 10 GHz, which can later be used for the development of radar-based MWI devices focused on the detection of CLNs. To the best of the authors’ knowledge, the proposed work consists of the first and unique realistic head and neck phantom generator available to all in the Supplementary Materials section, with the purpose of unifying data and results amongst research groups, following the FAIR principles for scientific research: Findable, Accessible, Interoperable, Reusable. Additionally, the generator was designed to cover all stages of research development with phantoms varying in complexity, number of tissues included, type of tissues included, and frequency range. The customization process is another strength of the designed generator as researchers have total freedom of choice regarding the MRI exam from which the phantom is derived and the features of the phantom. Accordingly, a multitude of phantoms can be designed from this generator in a short period of time. 

This paper is organized as follows. Section 2 details each stage of the process of building the 3D anthropomorphic phantom generator. Section 3 shows the interfaces of the generator which allow users to customize their phantoms. In addition, the current section depicts axial slices of the 3D phantom when tissues are included in the simplest phantom and when tissues are iteratively inserted in order to produce a more complex phantom. Section 4 discusses the organization and functioning of the phantom generator and its outputs and Section 5 highlights the versatility of the developed generator in constructing phantoms suited for the development process of a radar-based MWI device and also the broadening of its applicability to other research fields.

## 2. Materials and Methods

The 3D anthropomorphic phantom generator was designed using MATLAB^®^ R2018a. As inputs, the generator requires three matrices: (i) matrix with the binary masks of an MRI exam—Matrix of Binary Masks, (ii) the pre-processed MRI exam—MRI Matrix, and (iii) a matrix with the labels of the groups of tissues identified in the MRI exam—Matrix of Clusters. These inputs can be obtained at stage 0 of the process of building the phantom generator, which is detailed in [19,20]. A short summary of stage 0 is presented below for completeness. Stages 1, 2 and 3 of the phantom generator building process concern: stage 1) the selection of which biological tissues to include in the phantom as per tissues identified in the MRI exam; stage 2) insertion of skin and CLNs (synthetical tissues) in the phantom; and stage 3) the association of the realistic dielectric properties of the biological tissues to the tissues represented in the phantoms, respectively. Each one of these stages is detailed below.

### 2.1. Stage 0

Stage 0 comprises three main steps: 1) the selection of an MRI exam with high resolution where the anatomical structures are easily identified—the MRI exam used in this project was the FL3D T1 VIBE AXIAL FS-08594 sequence of subject TCGA-BA-5557, available on The Cancer Genome Atlas Head-Neck Squamous Cell Carcinoma (TCGA–HNSC) collection of The Cancer Imaging Archive (TCIA) [21], 2) data cleaning, which involved data Maximum–Minimum normalization, background subtraction with manually created binary masks and filtering techniques to remove noise and smooth sharp edges in MATLAB^®^, and finally, 3) the segmentation of the anatomical structures with K-means unsupervised clustering algorithm with a number of five clusters (*k* = 5), using the Statistics and Machine Learning toolbox from MATLAB^®^. The tested step 2) filtering techniques necessary to prevent the noise corruption of the images included: three linear LowPass (LP) filters—the ideal, Butterworth and Gaussian—which cover a range from very sharp (ideal) to very smooth filter functions (Gaussian) [22], and the non-linear median filter as it is very effective in the presence of Salt and Pepper noise [22], which is commonly present at MRI images [23]. Besides testing each filter individually, filters combinations were also attempted in order to obtain the best conjugation of filters characteristics which provided better quality images. The results of the filtering tests showed that both ideal and Butterworth filters resulted in images with several small-sized granularities, which compromise 3D-printed phantoms, median filters proved to be best for smoothing edges and Gaussian filters were very effective in removing noise. The combination of the last two, in that order, was proven as the best approach for data pre-processing since it allowed the best anatomical information retrieval. For segmentation purposes, four different widely used unsupervised clustering algorithms were tested: agglomerative Hierarchical Cluster Analysis (HCA), K-means clustering, BIRCH and DBSCAN [24]. In comparison with the other algorithms, the K-means with a *k* = 5 excelled in speed and good clustering quality.

**Figure 1:**
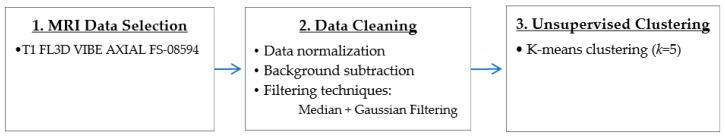
Schematics of the methodology pipeline followed to obtain the matrices required for the initialization of the 3D anthropomorphic phantom generator.

The three input matrices—Matrix of Binary Masks, MRI Matrix and Matrix of Clusters—were obtained as a result of step 2 and step 3. Figure 1 shows the adopted methodology pipeline to obtain the stage 0 matrices required for the initialization of the phantom generator.

The adopted pipeline allowed the identification of muscle and fat tissue, along with a highly heterogeneous combination of tissues, which we called mixed tissue. The mixed tissue represents an extremely complex area of compact tissues with different sizes, shapes and directions, which includes varying amounts of adipose tissue, loose connective tissue, muscle and small bones [25].

### 2.2. Stage 1—Biological Tissues

Initially, the 3D anthropomorphic numerical phantom derived from stage 0—the default phantom—is used to initialize the phantom generator. The default phantom contains biological tissues, such as fat, muscular and mixed tissue, each one segmented to a particular cluster (information provided by the Matrix of Clusters).

In stage 1, the user will be asked to specify the tissues to be incorporated in the phantoms. At this stage, the removal of the MRI-derived biological tissues provides simpler phantoms. Hence, when the user chooses not to include a certain stage 1 MRI-derived tissue, the volume segmented for that tissue is modelled as fat tissue. 

Firstly, the generator queries the user about the inclusion of the mixed tissue in the phantom. In this case, if the answer is affirmative, the voxels assigned to the mixed tissue cluster maintain their assignment to that cluster, otherwise the phantom is simplified and the voxels are labelled as fat tissue. Secondly, the generator prompts the user whether to include muscular tissue in the phantom. If affirmative, the muscle tissue cluster is maintained, otherwise the voxels corresponding to the segmented muscle cluster are assigned to the fat tissue cluster.

### 2.3. Stage 2—Synthetic Tissues

Stage 2 consists of the insertion of synthetic tissues for the tissues whose segmentation in stage 0 failed, namely skin tissue and CLNs. A second group of questions, concerning the inclusion of synthetic tissues will be asked at this stage. 

When the skin is selected and added to the phantom, a dedicated part of our algorithm generates a synthetic layer of skin which outlines the body boundary Its inputs are the Matrix of Binary Masks and the expected thickness of the skin layer, which is fixed to 1.4 mm according to [26]. At each slice of the transverse plane that divides the body into superior and inferior parts, this algorithm determines the position where the mask pixels change in value between 0, for background, and 1, for body region, and adds the synthetic layer of skin on the surface of the body region.

If the user decides to include CLNs, the generator will insert CLNs modelled with an oval shape, size ranging from 1.0 to 25.0 mm in its major axis, and two regions: an external region of perinodal adipose tissue, and an interior of the CLN itself [27]. In order to customize the phantom, features of the CLNs can be controlled in the generator—this includes controlling their number (for the MRI we used and determined a maximum of twelve CLNs in total and six per level, so as to guarantee that CLNs can be located in the chosen level and do not overlap regardless of their size), size, location and medical state, (i.e. healthy or malignant). The placement of the CLNs into the levels and the definition of the limits of each level—which is provided by the generator to the user—results from a procedure with several simplifications since the thresholds of the levels vary across slices, and also per patient. For the case in which the location of the CLNs is not specified by the user, the phantom generator will randomly attribute a location to each CLN.

### 2.4. Stage 3—Assignment of the Dielectric Properties and Save the Phantom

**Figure 2:**
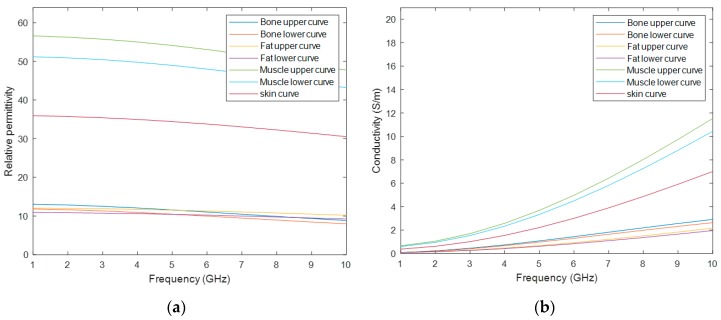
Dielectric properties curves considered to assign the dielectric properties to the tissues in the phantoms. The curves were obtained by using the 4-pole Cole–Cole formulation with the parameters given in [30]. (**a**) depicts the graph of the relative permittivity curves for bone, fat, muscle and skin tissues. (**b**) depicts the graph of the conductivity curves for the considered biological tissues.

As Cole–Cole models offer a suitable approach for representing the frequency variation of biological tissues at frequencies used for medical MWI, they were implemented at stage 3. In particular, the 4-pole Cole–Cole model, which is considered the best fitting technique as it describes the four relaxation mechanisms exhibited by biological tissues in a frequency range of 10 Hz to 100 GHz [28]. Initially, to assign dielectric properties to MRI-derived models, four dielectric properties curves for relative permittivity and conductivity were considered: a curve for skin, bone, fat and muscle tissue – the same tissues as those segmented or synthetically added in stage 0. In order to account for the dielectric differences within bone, fat and muscle tissue due to physiological processes, a dielectric variation of 5% with respect to the nominal property was incorporated [29]—except for skin. Thus, seven curves of the dielectric properties were obtained: one for the skin tissue, and two curves which limit the lower and upper bounds modelled for the bone, fat and muscle tissues. Although the curves cover a large range of frequency, only the range of interest for medical MWI (1-10 GHz) is depicted in Figure 2. The 4-pole Cole–Cole parameters for each curve are shown in Table A1 of Appendix A [30].

**Figure 3:**
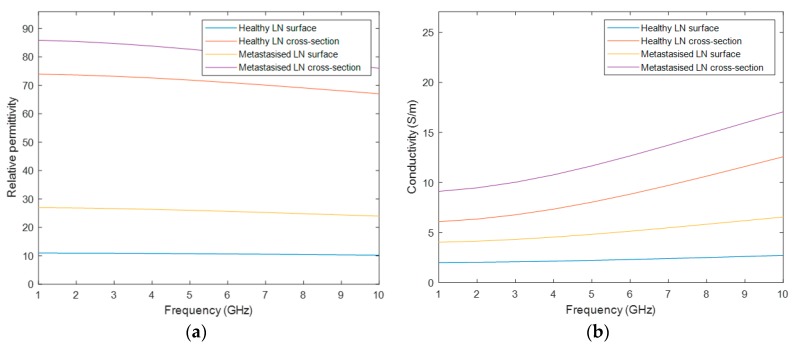
Dielectric properties curves considered to assign the dielectric properties to the Cervical Lymph Nodes (CLNs) inserted in the phantoms. The curves were obtained by using the 4-pole Cole–Cole formulation with the Debye parameters given by [31]. (**a**) depicts the graph of the permittivity curves of the cross-section and surface of the healthy and metastasized CLNs. (**b**) depicts the graph of the conductivity curves, for the cross-section and surface of the healthy and metastasized CLNs.

Healthy and malignant lymph nodes were modelled with a Debye model according to the values reported by Eleutério in [31], extrapolated from Cameron [32], as the 4-pole Cole–Cole parameters for these tissues are not available in the literature. The parameters used to model the LNs refer to the Debye model, which is a simplification of the Cole–Cole model, hence the Debye parameters were used to define the 1^st^ pole of the Cole–Cole model and the 2^nd^–3^rd^–4^th^ poles were assumed to be null in the 4-pole Cole–Cole model. The parameters are shown in Table A2 of Appendix A, and Figure 3 depicts the four curves of the dielectric properties of the healthy and metastasized LNs, on both the cross-section and surface. Similarly, the graphs in this figure only cover the frequency range of interest for medical MWI. 

In stage 3, the interface of the phantom generator allows the user to select a frequency between 1 to 10 GHz. Afterwards, the association of the realistic dielectric properties—relative permittivity and conductivity—of the biological tissues to the voxels of the phantom, for the user-specified frequency uses the curves of Figure 2 and Figure 3. The process of the assignment of the dielectric properties results in two matrices, one for each property, with the same dimension as the phantom. For each matrix, the voxels represent each dielectric property value, for the user-specified frequency, corresponding to each voxel of the anatomically realistic phantom. In order to obtain these matrices, these steps have to be followed: (1) For each cluster with a segmented tissue, the maximum and the minimum value of the intensity of the original MRI exam is determined; (2) These maximum and minimum intensity voxels are associated with the upper curve and the lower curve of the tissue, respectively; (3) The remaining voxels of the same cluster are linearly mapped to a value between the curves of that tissue. This methodology was inspired by Zastrow et al. [33]. As the mixed tissue consists of a mixture of multiple tissues, such as adipose, loose connective, muscle and bone tissues, the upper bound curve of the muscular tissue and the lower bound curve of the adipose tissue were set as the upper and lower curves of the mixed tissue, respectively. Hence, the dielectric properties variability of all tissues included in this cluster were covered.

After designing the phantom, users can choose whether they want to save the resulting files. In this case, the following matrices will be saved in .mat files: (1) the pre-processed MRI exam; (2) a matrix with the labelled tissues included in the phantom; (3) the matching conductivity matrix; and 4) the matching relative permittivity matrix.

## 3. Results

**Figure 4:**
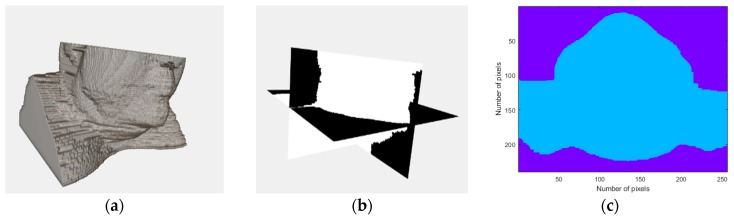
Visualizations of the simplest 3D head and neck phantom obtained from the developed phantom generator: a homogeneous phantom. (**a**) Realistic 3D rendering of the obtained phantom. (**b**) Scheme of the planes: coronal, sagittal and axial planes. (**c**) Corresponding axial plane of the generated phantom. Two regions are highlighted: the background (air) and the human body (homogenously modelled as fat tissue).

The developed phantom generator allows the inclusion of different types of tissues, independent of each other, which may be combined together in order to create multiple phantoms. The simplest 3D phantom which can be generated consists of the interior of the body (without skin) with only one tissue—fat tissue—which is the biological tissue with the lowest permittivity and conductivity values. In this case, no other tissues were inserted. Figure 4 shows a realistic 3D rendering of a phantom in (a), a scheme of the axes in (b), and the corresponding axial slice of the simplest phantom which can be obtained from the developed phantom generator in (c).

### 3.1. Stage 1

**Figure 5:**
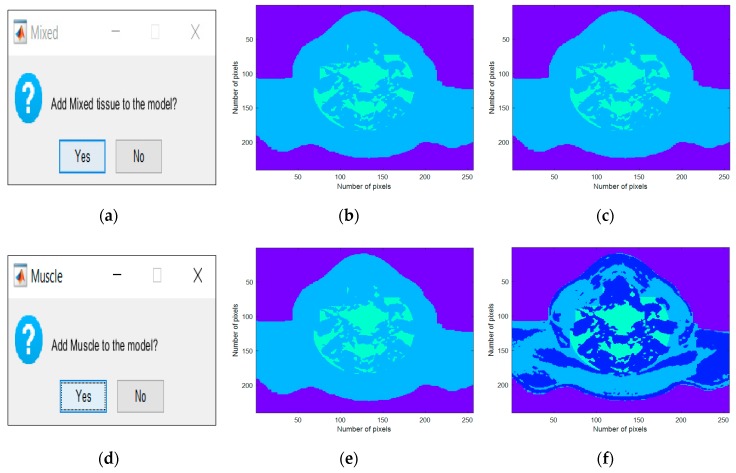
Interfaces created for the inclusion of the MRI-extracted tissues. (**a**) Generator interface which allows the users to include mixed tissue in the phantoms. (**b,c**) Axial slice of a 3D anthropomorphic head and neck phantom with fat and mixed tissues. (**d**) Interface which allows the user to insert muscle tissue in the phantom. (**e**) Axial slice of a 3D anthropomorphic head and neck phantom with muscle tissue. (**f**) Axial slice of a 3D anthropomorphic head and neck phantom which combines fat, mixed and muscle tissue.

Increasing the complexity of the generated phantoms consists of including more types of biological tissues present in the head and neck region. In stage 1, the interfaces created for the inclusion of the MRI extracted tissues are shown in the left column of Figure 5a,d, the center column of Figure 5b,e shows the case of the addition of each tissue alone and the right column of Figure 5c,f shows the process of combining tissues iteratively at each step of the process.

### 3.2. Stage 2

**Figure 6:**
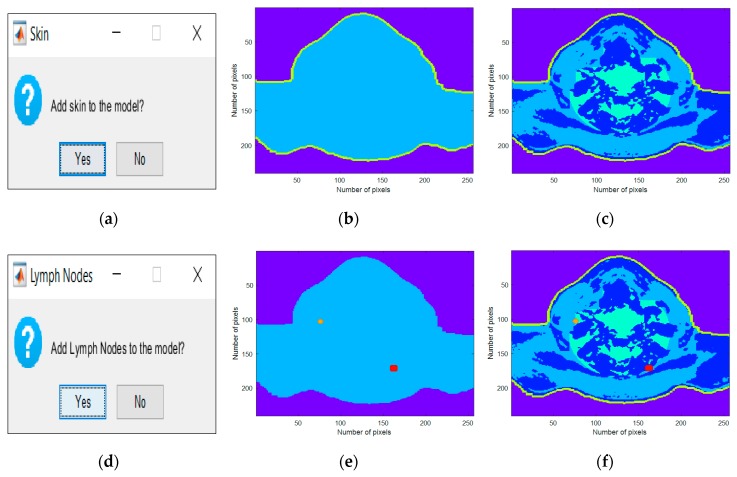
Interfaces created for the inclusion of the synthetic tissues. (**a**) Interface which allows the user to insert synthetic skin tissue in the phantom. (**b**) Axial slice of a 3D anthropomorphic head and neck phantom with skin tissue. (**c**) Axial slice of a 3D anthropomorphic head and neck phantom which combines fat, mixed, muscle and skin tissue. (**d**) Interface which allows the user to insert CLNs in the phantom. (**e**) Axial slice of a 3D anthropomorphic head and neck phantom with two CLNs: orange—with 1.5 mm major axis, healthy, and located on level II, and red—with 2.0 mm major axis, metastasized, and located on levels V. (**f**) Axial slice of a 3D anthropomorphic head and neck phantom which combines fat, mixed, muscle and skin tissues, and two CLNs.

The Stage 2 interfaces for the inclusion of synthetic tissues are shown in Figure 6a,d. The results of introducing each tissue alone and their combination are also represented in Figure 6b,c,e,f. As an example, two CLNs were inserted: a healthy CLN in orange, with a 1.5 mm major axis, located in level II, and a metastasized CLN in red with a 2.0 mm major axis, located in level V. 

**Figure 7:**
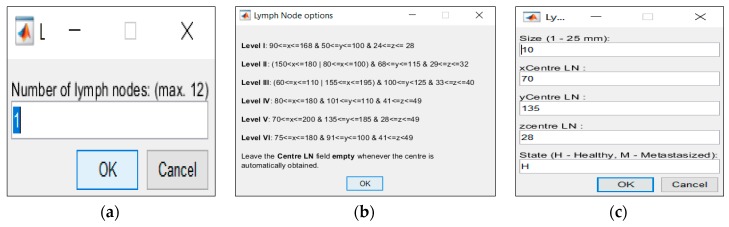
Interfaces created to modified CLNs. (**a**) Interface which allows the user to choose the number of CLNs to insert in the phantom. (**b**) Informative note which specifies the limits of the six levels of the CLNs. (**c**) Interface which allows the user to choose the size, location and medical state of each CLN.

These features may be modified in the interfaces shown in Figure 7a,c. Additionally, information regarding the limits of each levels is provided by the phantom generator in Figure 7b.

### 3.3. Stage 3

**Figure 8:**
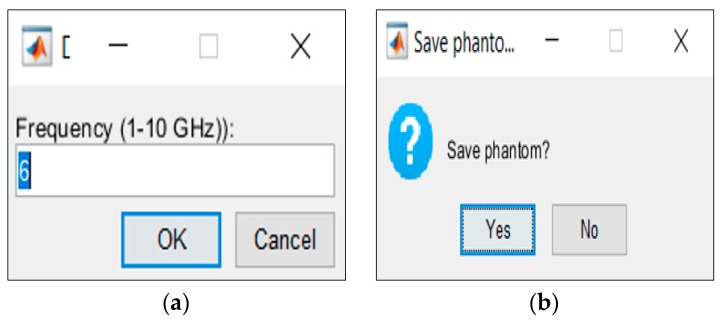
Stage 3 interfaces. (**a**) Generator interface which allow the users to choose the frequency value at which the phantom generator should interpolate the dielectric properties values of the biological tissues for a given frequency. (**b**) Interface which allows the user to save the phantom.

Stage 3 concerns the association of the dielectric properties of the biological tissues, for a user-specified frequency, to the voxel intensity of the MRI. Additionally, the user can save the final phantom. The interfaces which allow the user to choose the value of frequency and save the phantom are shown in Figure 8a,b, respectively.

## 4. Discussion

The developed phantom generator was constructed based on a building-block technique which allowed the inclusion of several independent types of biological tissues in a 3D anthropomorphic head and neck phantom—a unique tissue representing the whole-body volume. In this case, the fat tissue was considered the best choice to represent the overall human anatomy due to its lower values of relative permittivity and conductivity. From a simulation or experimental testing perspective, if the interior of the generated phantoms consists of tissues with low conductivity and relative permittivity, then the microwave radiation travels for longer with lower attenuation until it interacts with structures/regions with higher dielectric properties, and so more internal detail inside the phantom can be extracted.

Stages 1 and 2 allowed the inclusion of biological and/or synthetic tissues in the generated phantoms according to the user’s directions, including the addition of CLNs, whose features the users can customize. Stage 3 concerns the association of the relative permittivity and conductivity values of the biological tissues, for a user-specified frequency, given the voxel intensity of the MRIs.

Both Figure 5 and Figure 6 consisted of three columns: the left column depicted the interface which was created for the insertion of each tissue, the center column depicted the inclusion of each tissue type in the simplest generated phantom, and, finally, the right column represented the cumulative effect of adding each tissue type. These figures were obtained as an example of tissues combinations that can be inserted in the phantoms; many more phantoms with different features may be constructed from the developed 3D anthropomorphic phantom generator. The interfaces in Figure 5a,d permitted the inclusion of mixed and muscle tissues, respectively. The concept of mixed tissue derived from the simplification of the representation of very complex and compacted areas which included several biological tissues. In Figure 5b,c, it is noticeable that we considered the center region of the neck as mixed tissue separately from the main muscular groups. As a result, from the tissues considered in the mixed tissue, larger ranges of relative permittivity and conductivity were assigned to this area by setting the muscle tissue and the fat tissue curves as the upper and lower bound curves, respectively. A synthetic layer of skin with 1.4 mm may be inserted in phantoms through the interface in Figure 6a and, finally, CLNs can be included in the interface in Figure 6**d** and customized in interfaces of Figure 7a,c.

After the selection of the tissues to include in the phantoms, the association of the dielectric properties to those tissues is made at stage 3, for the frequency chosen at the interface in Figure 8a. 

This results in two matrices with the same dimensions as the phantom, one storing the relative permittivity values and another storing the conductivity values. These two matrices effectively represent 3D maps with the variation in the two dielectric properties of the tissues in the phantom. Finally, users can save the final phantom through the interface in Figure 8b. 

## 5. Conclusions

In this paper, we detailed the functioning of the proposed 3D anthropomorphic phantom generator which allows for the creation of customized phantoms of the head and neck region of the human body. The developed phantom generator was projected in order to give the user freedom of choice regarding the phantom level of complexity, the real anatomical tissues extracted from an MRI exam and the synthetic tissues to be included in the phantom, the features of the CLNs, and the frequency value at which the phantom generator should interpolate the dielectric properties of the included tissues.

As the functioning of the generator is quite intuitive and users can decide on the features they wish to project onto the phantoms, several combinations of characteristics can be designed in order to build phantoms matching the interests of the researchers. 

The resulting 3D anthropomorphic head and neck phantoms vary in complexity and in the type of tissues included, thus we expect them to have a great impact on the development process of MWI devices aimed at screening and diagnosing CLNs and, consequently, contribute to a non-invasive staging of the head and neck cancer. Besides, as the developed phantom generator is very versatile, its applicability may be extended to other research fields where head and neck phantoms are required. 

## Supplementary Materials

The following is available online at http://ibeb.ciencias.ulisboa.pt/raquel-conceicao: executable phantom generator program.

## Appendix A

**Table A1:** Parameters of the 4-pole Cole–Cole formulation from [30], in a frequency range from 10 Hz to 20 GHz, for the cervical tissues represented in the 3D anthropomorphic phantoms resulting from the developed phantom generator.

Tissues	ε_∞_	(σ_s_) [S/m]	∆ε_1_	∆ε_2_	∆ε_3_	∆ε_4_	α_1_	α_2_	α_3_	α_4_	τ_1_ [ps]	τ_2_ [ns]	τ_3_ [μs]	τ_4_ [ms]
Bone	2.500	0.020	10.00	180.0	5 × 10^3^	1 × 10^5^	0.200	0.200	0.200	0.000	13.263	79.577	159.155	15.915
Fat	2.500	0.035	9.00	35.00	3.3 × 10^4^	1 × 10^7^	0.200	0.100	0.050	0.010	7.958	15.915	159.155	15.915
Muscle	4.000	0.200	50.00	7000	1.2 × 10^6^	2.5 × 10^7^	0.100	0.100	0.100	0.000	7.234	353.678	318.310	2.274
Skin	4.000	0.000	32.00	1100	0.000	0.000	0.000	0.200	0.200	0.200	7.234	32.481	159.155	15.915

**Table A2:** Parameters of the Debye model for LNs from [31] suitable to extract dielectric properties at a center frequency of 7.5 GHz, applied to a 1-pole Cole–Cole model.

Tissues	ε_∞_	(σ_s_) [S/m]	∆ε_1_	α_1_	τ_1_ [ps]
**Healthy LN surface**	8.00	2.00	3.00	0.200	9.24
**Healthy LN cross-section**	47.00	6.00	27.00	0.200	9.40
**Metastasized LN surface**	17.00	4.00	10.00	0.100	10.47
**Metastasized LN cross-section**	55.00	9.00	31.00	0.000	11.00
